# Synonymous variants in the *ATP6AP2* gene may lead to developmental and epileptic encephalopathy

**DOI:** 10.3389/fneur.2023.1320514

**Published:** 2024-01-11

**Authors:** Yan Liang, Lin Wan, Huimin Yan, Xinting Liu, Jing Zhang, Gang Zhu, Guang Yang

**Affiliations:** ^1^Department of Pediatrics, Seventh Medical Center of PLA General Hospital, Beijing, China; ^2^Department of Pediatrics, First Medical Centre, Chinese PLA General Hospital, Beijing, China; ^3^Medical School of Chinese People’s Liberation Army, Beijing, China

**Keywords:** *ATP6AP2*, *ATP6AP2*-related DEE, developmental and epileptic encephalopathy, synonymous variants, exon jumping

## Abstract

**Objective:**

To the literature, variants in the *ATP6AP2* gene may cause abnormal nervous system development and associated neurological symptoms.

**Methods:**

We report a patient with developmental and epileptic encephalopathy (DEE) carrying an ATP6AP2 c.858G > A (p.Ala286=) synonymous variant. In addition, an overview of reported patients with the same variant were collected and summarized to compare our findings.

**Results:**

The patient started experiencing tonic seizures at 3.5 months of age, and magnetic resonance imaging (MRI) indicated impaired brain white matter development and reduced left hippocampal volume. Furthermore, electroencephalography showed multifocal interictal epileptiform discharges. Treatment with various anti-seizure medications yielded unsatisfactory results, and the disorder eventually developed into epileptic spasms. An *in vitro* splicing assay for the *ATP6AP2* gene mRNA revealed that the variant caused a deletion in exon 8 and a corresponding protein truncation. A review of previously reported *ATP6AP2*-related DEE patients found that synonymous variants in the *ATP6AP2* gene can cause early DEE onset, progressive changes in early-life MRI, and exon skipping in all *ATP6AP2*-related DEE patients.

**Significance:**

We found that synonymous variants in *ATP6AP2* may have significant pathogenicity and are highly correlated with DEE. Due to increased isoform production, *ATP6AP2* synonymous variants may cause nervous system developmental disorders by competitively reducing the generation of full-length transcripts, resulting in defects in *ATP6AP2*-related physiological processes.

## Introduction

The *ATP6AP2* gene is located on Chromosome Xp11.4 and encodes a type I transmembrane protein that is primarily expressed in the brain, heart, and placenta, followed by the kidneys and pancreas (1). Previous research has focused mainly on its role as a renin receptor involved in blood pressure regulation and the renin–angiotensin system. Recent studies suggest that variants in the *ATP6AP2* gene may cause abnormal nervous system development and corresponding neurological symptoms, including Parkinson-like manifestations, global developmental delay, and epilepsy, with significant phenotypic differences among individuals carrying different variants.

The Hedera type of X-linked syndromic intellectual developmental disorder (MRXSH) is an infantile-onset syndrome characterized by global developmental delay, progressive cognitive decline, abnormal movements, and epilepsy. It was first reported by Hedera et al. (2) in 2002 in a family with seven male members exhibiting the aforementioned phenotype. Genetic analysis suggests that abnormalities in Xp21.1-p11.4 may be the predominant cause of this disease. Subsequently, Ramser et al. (3) investigated the family further and found that synonymous variants affecting *ATP6AP2* gene splicing may be responsible for the disease phenotype in this family.

Developmental and epileptic encephalopathies (DEEs), a heterogeneous group of disorders with nongenetic and genetic etiologies, are characterized by severe epileptic seizures and electroencephalography (EEG) abnormalities with a background of developmental impairment that tends to worsen as a consequence of epilepsy (4).

Previous studies have shown that patients with *ATP6AP2*-related MRXSH usually exhibit a significant DEE phenotype. When compared to Parkinson’s symptoms, reports of DEE, a severe developmental illness, are comparatively infrequent and mostly case reports. Herein, we report a patient with DEE associated with an *ATP6AP2* synonymous variant, functionally validate its pathogenicity, and review previously reported *ATP6AP2*-related DEE cases to clarify the potential association between genotype and DEE phenotype.

## Methods

### Patients

The patients with confirmed *ATP6AP2* variant accompanied by developmental delay and epilepsy were enrolled from the First Medical Center of PLA General Hospital. Detailed clinical information was collected, including clinical manifestations, history of epilepsy, physical examination, treatment, EEG, and magnetic resonance imaging (MRI).

### WES analysis

Genomic DNA was extracted from the peripheral blood of the patients and their parents for trio whole-exome sequencing (Trio-WES). In accordance with the requirements of Research Ethics Board at First Medical Center of PLA General Hospital, informed consent was obtained from the patient’ parents for participation in Trio-WES and subsequent Sanger sequencing.

According to the manufacturer’s instructions, the QIAamp DNA Blood Mini Kit (Qiagen GmbH, Hilden, Germany) was utilized to extract and purify 1 μg of DNA from a 200 μL blood sample. The DNA libraries were constructed using a polymerase change reaction (PCR)-free method. Next-generation sequencing was employed for mutation screening. The NanoWES Human Exome platform (Berry Genomics Corporation, Beijing, China) was used for whole-exome sequencing, which was performed on an Illumina NovaSeq 6000 instrument (Illumina, San Diego, United States). Single-nucleotide polymorphisms (SNPs), insertions or deletions (InDels), and splicing events (SPIDEX, dbscSNV, spliceAI, and NetGene2) were identified through bioinformatics analysis. The pathogenicity of variants was assessed following the standards and guidelines of the American College of Medical Genetics and Genomics (ACMG) (5). The sequencing reads were aligned to the human reference genome (hg38/GRCh38).

### Minigene analysis

To investigate the potential splicing effects resulting from the c.858G > A mutation, an *in vitro* minigene splicing assay was conducted. The minigene regions encompassing ATP6AP2 exon 7–9 and intron 7–8 of the *ATP6AP2* gene were amplified from control gDNA using the forward primer ATP6AP2-F (5′-GATATACACTGTTTGAGATGAGGA-3′) with the BamHI restriction site and the reverse primer ATP6AP2-R (5′-TCATCACTGGCAAAGCACAC-3′) with the XhoI restriction site. The amplified products were cloned into the pMini-CopGFP vector (Beijing Hitrobio Biotechnology Co., Ltd.) using the ClonExpress II One Step Cloning Kit (Vazyme, Nanjing, China). The wild-type plasmid was confirmed by Sanger sequencing, while the mutant fragments were generated using ATP6AP2-MT-F (5′-GATATACACTGTTTGAGATGAGGA-3′) and ATP6AP2-MT-R (5′-TCATCACTGGCAAAGCACAC-3′) mutagenesis primers. The mutant plasmid was validated by Sanger sequencing. Selected plasmids were prepared for subsequent transfection. Human embryonic kidney 293 T (HEK293T) cells were cultured in Dulbecco’s modified Eagle’s medium supplemented with 10% fetal bovine serum (HyClone) and incubated at 37°C with 5% CO_2_. The recombinant plasmids were transiently transfected into HEK293T cells using Lipofectamine 2000 (Invitrogen) following the provided instructions. Total RNA was extracted from cells cultured for 48 h using TRizol reagent (Cowin Biotech Co.). Reverse transcription-polymerase chain reaction (RT-PCR) was performed using the primer pair MiniRT-F (5′-GGCTAACTAGAGAACCCACTGCTTA-3′) and MiniRT-R (5′-TCAATCCATTCGAATCTTCTGGTTTG-3′). PCR fragments were analyzed by agarose gel electrophoresis, and isoforms were determined by Sanger sequencing.

## Results

### Case presentation

**Figure 1:**
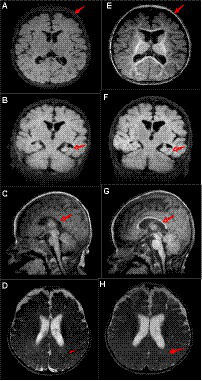
Patient’s brain MRI. At 4 months of age, **(A)** the T1 axial view shows brain atrophy; **(B)** the T1 coronal view shows reduced left hippocampal volume; **(C)** the T1 sagittal view shows a slightly thin corpus callosum; and **(D)** the T2 axial view shows impaired myelin development. At 14 months of age, **(E)** the T1 axial view shows widened extracerebral spaces; **(F)** the T1 coronal view shows reduced left hippocampal volume; **(G)** the T1 sagittal view shows a slightly thin corpus callosum (arrows, similar to **C**); and **(H)** the T2 axial view shows impaired myelin development (arrows, similar to **D**). Compared to 4 months of age, at 14 months, there is deepening of the cerebral sulci, increase relative volume of the lateral ventricles, and a reduction in the left hippocampal volume similar to before.

A male patient from non-consanguineous parents was born at term. He experienced epilepsy at 3.5 months of age, which was characterized by right-sided focal tonic seizures. Brain MRI revealed a slightly thin corpus callosum (Figures 1A–D). EEG showed no hypsarrhythmia but multifocal spike and spike-slow wave discharges, predominantly in the left hemisphere. Subsequently, the patient was treated with oxcarbazepine, but seizure control was not achieved. At 4.5 months, his seizure pattern changed to epileptic spasms, and despite treatment with adrenocorticotropic hormone (did not try prednisolone PO) and various anti-seizure medications (oxcarbazepine 35 mg/kg/d, vigabatrin 100 mg/kg/d, topiramate 5 mg/kg/d, lamotrigine 4 mg/kg/d, clobazam 1 mg/d, perampanel 2 mg/d, zonisamide 6 mg/d), they remained uncontrolled. At 14 months, he still experienced epileptic spasms, and his EEG revealed bilateral multifocal spikes and spike-slow wave discharges and there was no hypsarrhythmia. Comparing the MRI results at 4 months, the subarachnoid space was widened, the left hippocampus volume was reduced, and the delay in brain white matter myelination was significant (Figures 1E–H). A *de novo ATP6AP2* heterozygous synonymous variant, NM_005765:c.858G > A (p.A286A), was identified through family whole-exome sequencing. At 20 months, the patient exhibits global developmental delay, only being able to smile and lift his head without reaching other psychomotor developmental milestones, such as sitting independently, following instructions, or engaging in verbal communication.

### Variant results in the down-regulation of RNA and protein

**Figure 2:**
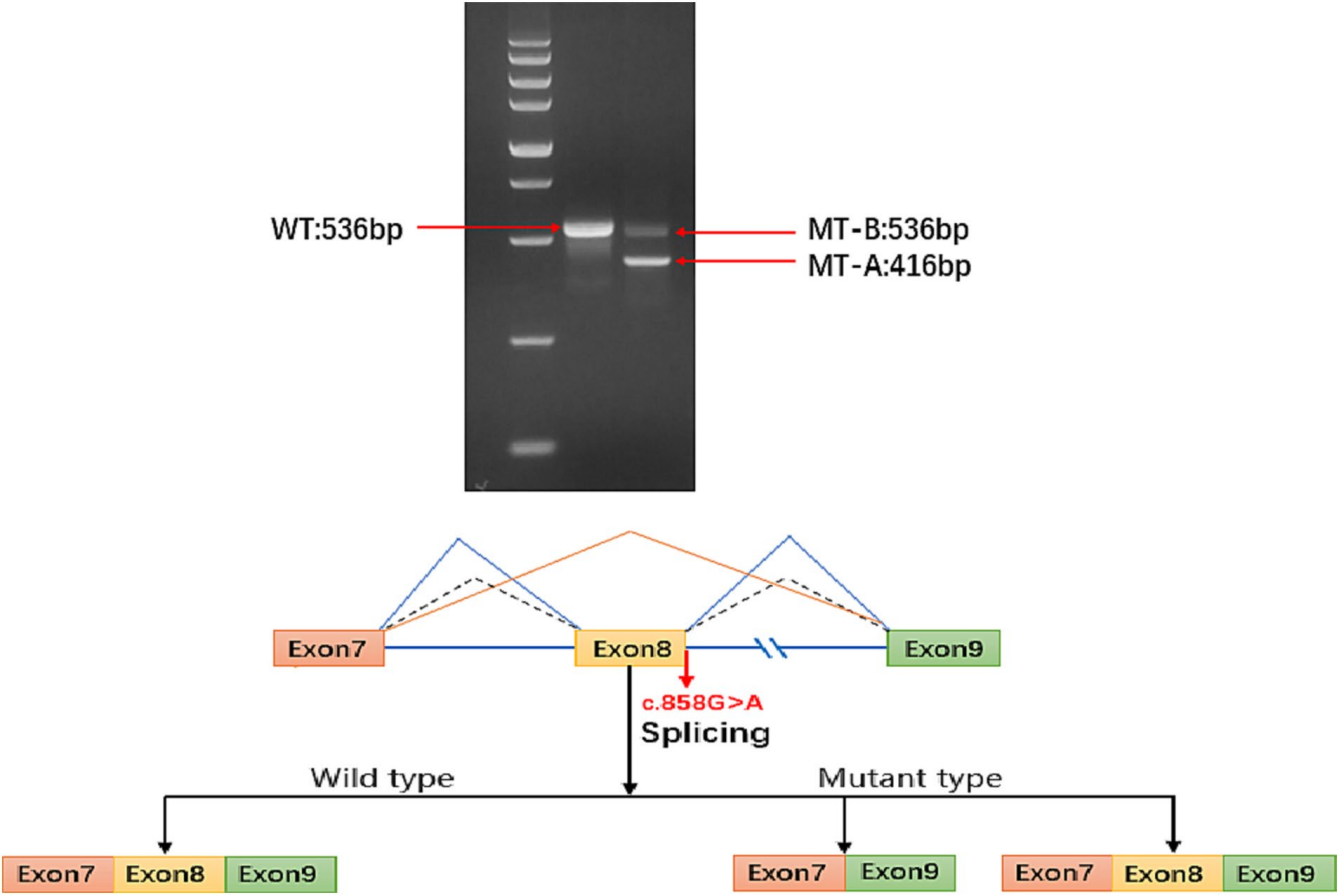
Electrophoresis results show that the sequence length of *ATP6AP2* PCR-amplified wild-type (WT) mRNA is 536 bp, while the majority of the mutant-type (MT) mRNA sequence length is 416 bp, with a small portion of MT transcribed into a truncated mRNA product with a length of 536 bp. In the mature mRNA, there is a 120 bp sequence deletion in exon 8, represented as NM_005765: c.739_858del. In the absence of nonsense-mediated mRNA decay (NMD), the protein deletion forms a truncated protein, represented as p.Phe247_Ala286del. The blue solid line represents the two splicing patterns of the MT type exons (exon 7 and exon 9, and exon 7, exon 8 with exon 9). The blue dashed line represents the sole splicing pattern of the WT type, which includes exon 7, exon 8, and exon 9.

The *ATP6AP2* synonymous variant c.858(exon8)G > A (p.A286A) is classified as likely pathogenic according to the American College of Medical Genetics and Genomics criteria (PS2 + PM2 + PP3). Considering that some synonymous variants may not affect gene function, as reported in previous studies (6), we used minigene splice assays to construct wild-type and mutant *ATP6AP2* minigene plasmids. These plasmids were transfected into 293 T cells, and RNA was extracted, reverse-transcribed into cDNA, and amplified by PCR. Furthermore, PCR fragment size and sequencing results were analyzed. The results showed that there were two kinds of mRNA products been produced. The first product had 120 bp (c.739_858del.) sequence deletion in exon 8, resulting in a truncated protein without nonsense-mediated mRNA decay. The second product had a splice pattern consistent with the wild-type, containing intact exons 7, 8, and 9 (Figure 2).

## Discussion

The *ATP6AP2* gene is involved in Wnt signaling, tissue RAS activation, and tyrosine phosphorylation signal transduction (7). Skipping exons seems to be the pathogenic mechanism resulting from *ATP6AP2* gene synonymous pathogenic variants as illustrated by the following example cases. In addition to the full-length isoform, previous studies have identified 10 different alternatively spliced isoforms of *ATP6AP2* in various human tissues, including the brain, but at extremely low levels (8). According to Korvatska et al. (8), the proportion of spliced isoforms in brain tissue may be 10 times higher than in peripheral blood. Additionally, exon 4 skipping is increased in the synonymous variant c.345C > T (p.S115S), leading to a 90-fold increase in the level of isoforms lacking exon 4 in peripheral blood, which compete with the full-length transcript, resulting in reduced full-length transcript levels and autophagy abnormalities that caused neurological symptoms. Similarly, Edelman et al. (9) found that c.168 + 6 T > A decreased the production of full-length transcripts by increasing exon 2 skipping, leading to neurological symptoms. Consistent with previous research, our case resulted in two transcripts by increasing exon 8 skipping, leading to a decrease in full-length transcripts and corresponding symptoms.

The synonymous mutation ultimately affects the level of the normal full size *ATP6AP2* isoform and will compete with the pre-mRNA of normal *ATP6AP2* subtype at the transcriptional level, resulting in a decrease in the production of normal isoforms. Additionally, the mutated isoforms have a shorter half-life, leading to an overall reduction in ATP6AP2 protein levels and consequently contributing to the occurrence of the disease.

Reported *ATP6AP2*-related DEE patients have variants causing exon skipping, primarily in exons 2 and 4. For example, Ramser et al. (3) reported a patient with the DEE phenotype due to the *ATP6AP2* c.321C > T synonymous variant causing exon 4 skipping, significant developmental delay, and early-life epilepsy. Hirose et al. (10) reported a patient with developmental delay and infantile epilepsy due to an *ATP6AP2* intronic variant c.301-11_301-10del that increased exon 4 skipping. Gupta et al. (11) recently reported a patient with typical developmental delay and infantile epilepsy due to the intronic variant c.168 + 6 T > A causing exon 2 skipping. In our *ATP6AP2*-related DEE patient, the synonymous variant led to exon 8 skipping. Thus, exon skipping appears to be a common feature in all *ATP6AP2*-related DEE patients.

At the 14-month MRI re-examination of our patient, the subarachnoid space was significantly widened compared to that at 4 months, the left hippocampus volume was reduced further, and myelination delay was evident, all of which indicate the possibility of brain atrophy. Significant global cerebral hemisphere atrophy was also observed in a previously reported *ATP6AP2*-related DEE patient by Gupta et al. (11) Furthermore, Hirose et al. (10) reported rapid brain atrophy progression in another *ATP6AP2*-related DEE patient. Therefore, we believe that the progressive brain atrophy observed in brain MRI may be a characteristic imaging feature of *ATP6AP2*-related DEE; however, not unique to this disorder as other genetic-metabolic disorders could present a similar finding.

Our study indicates that synonymous variants in *ATP6AP2* may have significant pathogenicity and are highly correlated with DEE. These variants may also lead to nervous system developmental disorders due to the increased production of isoforms, competitively reducing the generation of full-length transcripts, and defects in *ATP6AP2*-associated physiological processes. This finding will help to further characterize variants of uncertain significance that have been reported in ClinVar or other databases and relabel into pathogenic closing diagnostic chapters in some families dealing with the uncertainty of lack of diagnosis.

## Data availability statement

The datasets presented in this article are not readily available because of ethical and privacy restrictions. Requests to access the datasets should be directed to the corresponding author/s.

## Ethics statement

The studies involving humans were approved by the First Medical Center of PLA General Hospital. The studies were conducted in accordance with the local legislation and institutional requirements. Written informed consent for participation in this study was provided by the participants' legal guardians/next of kin. Written informed consent was obtained from the individual(s), and minor(s)' legal guardian/next of kin, for the publication of any potentially identifiable images or data included in this article.

## Author contributions

YL: Writing – original draft. LW: Writing – original draft. HY: Resources, Writing – review & editing. XL: Data curation, Writing – review & editing. JZ: Data curation, Writing – review & editing. GZ: Validation, Writing – review & editing. GY: Writing – review & editing.
